# Control of bleeding by silk ligation and diathermy coagulation during tonsillectomy: A comparison of efficacy of the two techniques in the first 24 hours after surgery

**DOI:** 10.12669/pjms.314.7445

**Published:** 2015

**Authors:** Khurshid Anwar, Rafiq Ahmad, Muneeb Khan

**Affiliations:** 1 Khurshid Anwar, Senior Registrar, Department of ENT and Head & Neck Surgery, Hayatabad Medical complex, Peshawar, Pakistan; 2 Rafiq Ahmad, Associate Professor, Department of ENT and Head & Neck Surgery, Khalifa Gul Nawaz Teaching Hospital Bannu, Pakistan; 3 Muneeb Khan, Trainee Medical Officer, Dept. of ENT and Head & Neck Surgery, PGMI HMC, Peshawar, Pakistan

**Keywords:** Tonsillectomy, Diathermy, Suture Ligation, Haemostasis, Primary haemorrhage

## Abstract

**Objective:**

To assess and compare the relative efficacy of silk ligation and diathermy coagulation techniques in controlling bleeding during tonsillectomy in the first 24 hours.

**Methods:**

This prospective study was conducted at the Department of ENT, Khalifa Gul Nawaz Teaching Hospital, Bannu and this department related consultants’ private clinics from January 1, 2012 to December 31, 2014. The study included 180 cases. All patients included were having history of recurrent, acute tonsillitis, with more than 6–7 episodes in one year, five episodes per year for two years, or three episodes per year for three years. All the surgeries were performed by dissection method. Haemostasis during the procedure was secured by either ligation with silk 1 or using diathermy. The results were analyzed using SPSS 16.0 for windows.

**Results:**

A total of180 cases were included in the study. The ages of the patients ranged from 5 to 40 years with the mean age of 15.56 years and a std.deviation of +/- 8.24. The male to female ratio was 1.25:1. The number of hemorrhages occurring was greater in the ‘diathermy coagulation’ group as compared to the ‘silk ligation’ group. However, the observed difference was statistically insignificant (p >.05).

**Conclusion:**

Primary haemorrhage occurring during tonsillectomy is a serious threat and control of bleeding during the procedure should therefore be meticulous. Both suture ligation and coagulation diathermy for control of bleeders during the procedure by dissection method are equally effective.

## INTRODUCTION

Tonsillectomy has been practiced for 2,000 years, with varying popularity over the centuries. Aulus Cornelius Celsus (25–50 AD) described a procedure whereby using the finger to separate the tonsils from the neighboring tissue before they were cut out. Galen (121–200 AD) was the first to advocate the use of the surgical instrument known as the snare. In the 7th century Paulus Aegineta (625–690 AD) described a detailed procedure for tonsillectomy, including dealing with the inevitable post-operative bleeding. The procedure saw disapproval in the Middle Ages. Scottish physician Peter Lowe in 1600 AD introduced the cold steel technique including the snare, the ligature, and the excision.^1^

The commonest indications for tonsillectomy in children are recurrent episodes of incapacitating acute tonsillitis that result in significant loss of school or work.^2^ Repeated attacks (3–4 per year for 2–3 years) of true acute tonsillitis are a definite indication for adult tonsillectomy.^3^ Today, tonsillectomy is one of the most commonly performed surgical procedures the world over and is usually safe and simple. It has been regarded as a major surgery because of its known postoperative haemorrhage and complications due to anaesthesia.^4^

Primary hemorrhage occurring <24 hour after surgery remains the most serious complication of tonsillectomy. In Norfolk and Norwich University Hospital, Norwich, UK Bennett AM and colleagues performed a meta analysis of all adult and paediatric tonsillectomy studies giving the absolute number and timing of all primary haemorrhages found that 93% of post operative bleeding in tonsillectomy occurred in the first 8 hours after surgery.^5^ Literature reviews suggest primary hemorrhage rates of 1-3% and secondary hemorrhage rates of 0.15-6.4%.^6^

Dissection tonsillectomy with haemostasis performed with or without suture ligation is the standard technique. Newer methods like the use of harmonic scalpel, coblation and Lasers have been found to be safe and efficient, reducing perioperative blood loss and operative time as compared to the older traditional methods. However, their cost and availability limit their use. This study aimed to determine the efficacy of bleeding control during tonsillectomy by suture ligation and the use of diathermy in our setting.

## METHODS

The study included 180 cases undergoing tonsillectomy during January 1, 2012 to December 31, 2014. Sample size was calculated using 40% proportion of tonsillectomy, 95% confidence level and 6.8% margin of error under WHO software for sample size determination.

### Study Design

It was a prospective and comparative study using the non-probability convenience sampling technique.

### Inclusion Criteria

1. Patients belonging to both the sexes.
2. Age 5 years and above.
3. When indication for surgery was chronic tonsillitis alone.

### Exclusion Criteria

1. Patients with bleeding diathesis.
2. Patients in whom both suture ligation and diathermy was used for haemostasis.
3. Abscess tonsillectomy and patients with episode of acute tonsillitis.
4. Patients undergoing adenotonsillectomy.

### Data Collection Procedure

Ethical approval for the study was obtained from the institutional ethical committee. Informed consent was obtained in all cases regarding the surgical procedure and inclusion in the study.

A detailed history was taken. All patients included were having history of recurrent, acute tonsillitis, with more than 6–7 episodes in one year, five episodes per year for two years, or three episodes per year for three years. All the patients were admitted to the ward and investigated to determine their fitness for general anaesthesia and the procedure. Haemoglobin level, viral screening and coagulation profile was tested in all the patients. All tonsillectomies were carried out by dissection method under general anaesthesia using propofol for induction and halothane for maintenance.

Patients were assigned on alternate basis into two categories with respect to mode of haemostasis; (1). By suture ligation and (2). Using diathermy. Suture ligation was carried out with silk 1 and diathermy used as bipolar or monopolar on coagulation setting. Complete haemstasis was achieved in both the groups. Patients in whom both silk ligation and suturing had to be carried out were excluded from final analysis. Patients were recovered and observed in the recovery room and in the ward by trained technical and nursing staff. Vital signs were monitored every 15 minutes in the recovery room and half hourly for the first two hours and then two hourly for the first 8 hours in the ward. Any excessive swallowing, bleeding from the corner of mouth, difficulty in breathing, nausea and vomiting noted and recorded on the chart.

Patients were examined by the consultant on discharge from the recovery room and 4-5 hours later in the ward at the end of the theatre hours. Patients were observed for 24 hours in the ward. Haemetemesis after complete recovery and clot adhering to the tonsillar fossa(e) were considered positive for bleeding. Blood stained saliva in the absence of rapid collection of blood for the first 12 hours was taken as normal.

All the data regarding age, gender, occurrence of haemorrhage in both the groups was recorded on a proforma. The data was analyzed using SPSS 16.0 for windows. Descriptive statistics like mean +/- standard deviation were calculated for quantitative variables like age and duration of symptoms. Frequency and percentages were calculated for categorical variables like gender, haemorrhage. The frequency of haemorrhage in the first 24 hours was compared in the two groups and the relative safety of day case tonsillectomy was inferred from the results obtained.

## RESULTS

A total of180 cases were included in the study. All the patients presented with chronic tonsillitis. The ages of the patients ranged from 5 to 40 years with the mean age of 15.56 years and a std.deviation of +/- 8.24. There were 100 males and 80 female patients. The male to female ratio was 1.25:1.The frequency of primary haemorrhage has been shown in various age groups with the maximum number occurring in the 31+ age group as depicted in Table-I. The gender wise frequency of hemorrhage was more in males as compared to females (Table-II). The number of hemorrhages occurring was greater in the ‘diathermy coagulation’ group as compared to the ‘silk ligation’ group as shown in Table-III. However, chi- square test and the p-value showed that there was no significant difference in the two techniques with respect to control of haemorrhage in the two groups (p >.05) as shown in Table-IV.

**Table-I:** Age-wise occurrence of haemorrhage in patients.

			Post op Bleeding		Total
			Yes	No	
Age (in years)	<= 10	Count	0	74	74
		% within Age (in years)	0.0%	100.0%	100.0%
	11 - 20	Count	0	60	60
		% within Age (in years)	0.0%	100.0%	100.0%
	21 - 30	Count	1	38	39
		% within Age (in years)	2.6%	97.4%	100.0%
	31+	Count	3	4	7
		% within Age (in years)	42.9%	57.1%	100.0%
Total		Count	4	176	180
		% within Age (in years)	2.2%	97.8%	100.0%

**Table-II:** Gender wise frequency of Primary haemorrhage.

			Post op Bleeding		Total
			Yes	No	
Gender of Patients	Male	Count	3	97	100
		% within Gender of Patients	3.0%	97.0%	100.0%
	Female	Count	1	79	80
		% within Gender of Patients	1.2%	98.8%	100.0%
Total		Count	4	176	180
		% within Gender of Patients	2.2%	97.8%	100.0%

**Table-III:** Post op Bleeding * Bleeding Control Cross tabulation.

			Bleeding Control		Total
			Silk Ligation	Diathermy Coag	
Post op Bleeding	Yes	Count	1	3	4
		% within Bleeding Control	1.1%	3.3%	2.2%
	No	Count	89	87	176
		% within Bleeding Control	98.9%	96.7%	97.8%
Total		Count	90	90	180
		% within Bleeding Control	100.0%	100.0%	100.0%

**Table-IV:** Statistical Significance.

	Value	df	Asymp. Sig. (2-sided)	Exact Sig. (2-sided)	Exact Sig. (1-sided)
Pearson Chi-Square	1.023^a^	1	0.312		
Continuity Correction^b^	0.256	1	0.613		
Likelihood Ratio	1.069	1	0.301		
Fisher’s Exact Test				0.621	0.310
Linear-by-Linear Association	1.017	1	0.313		
N of Valid Cases^b^	180				

a . 2 cells (50.0%) have expected count less than 5. The minimum expected count is 2.00.

b . Computed only for a 2×2 table.

## DISCUSSION

Post-tonsillectomy hemorrhage remains the most serious and even fatal complication of tonsillectomy.^7^ Minor bleeding does not require any active measures but major bleeding necessitates control of hemorrhage under general anaesthesia in the operation theatre. Haemostasis is usually secured by ligating the bleeders or coagulating them by diathermy, or by a combination of both of these. Primary hemorrhage is generally considered to be related to surgical technique whereas factors that influence wound healing contribute to secondary hemorrhage. Mortality from bleeding is 2 in 10,000 tonsillectomies.

Most cases of fatal postoperative bleeding occur within the first 24 hours after surgery.^8^ Recently published rates for primary hemorrhage within the first 24 hours after tonsillectomy vary from 0.3% to 2.1%.^9^ Whereas some other studies put this figure to be as high as 1.8% to 4%.^10^ In our study we found 2.2% of patients who suffered a significant reactionary haemorrhage. This was higher in the age group 31 and above possibly because of recurrent episodes of inflammation and fibrosis leading to more trauma during dissection.

A similar study conducted at a tertiary care hospital of Peshawar, Arif Raza Khan and colleagues reported no difference at all in the rate of primary hemorrhage (6.66%) whether haemostasis was achieved using bipolar diathermy or silk ligation.^11^ In contrast, Adel S and colleague in Iraq, in their study noted primary bleeding in 6 patients (2.4%) with bipolar diathermy haemostasis as compared to 13 patients (5.2%) with silk ligation.^12^ In a larger series of 1500 modified diathermy technique studied in Karachi, Rafique Gooda and colleagues observed only two patients with reactionary haemorrhage necessitating return to theatre.^13^ Shivkumar and colleagues in India in a study of 100 similar cases found equal frequency of primary haemorrhage and concluded that the Incidence of reactionary haemorrhage was same using electrocautery & ligation as method of haemostasis.^14^ In another study conducted by P.K.Moonka, diathermy failed to achieve adequate haemostasis in 4 out of 188 cases & Ligation was unsuccessful in 6 out of 188 cases. He concluded that bipolar diathermy is equally as effective as ligation in control of haemorrhage.^15^

## CONCLUSION

Primary haemorrhage occurring during tonsillectomy is a serious threat and control of bleeding during the procedure should therefore be meticulous. In our setting, tonsillectomy by dissection method is routinely performed. Both suture ligation and coagulation diathermy for control of bleeders during the procedure performed by this method are equally effective.
